# The effect of tennis on male bone mineral density: A meta-analysis

**DOI:** 10.1371/journal.pone.0328636

**Published:** 2025-08-25

**Authors:** Huang Huiwen, Huang Ju, Fu Dong, Fu Yan, Wang Tao

**Affiliations:** 1 Chengdu Physical Education University, Chengdu, China; 2 Hope College, Southwest Jiaotong University, Chengdu, China; 3 School of Physical Education, Southwest Minzu University, Chengdu, China; 4 School of Physical Education (Campus), Zhengzhou University, Zhengzhou, China; VA Loma Linda Healthcare System, UNITED STATES OF AMERICA

## Abstract

**Background:**

As an asymmetrical sport, tennis positively influences the increase of bone mineral density (BMD) in adolescents. However, most current studies focus on specific body regions, there is a lack of systematic analysis of the impact of tennis on BMD in various parts of the body. Therefore, this study aims to systematically assess the impact of tennis on BMD in different areas of the body.

**Objective:**

This study aimed to systematically evaluate the effects of tennis on BMD in males.

**Methods:**

Comprehensive search was conducted across databases including Web of Science, PubMed, CNKI, and Embase to identify high-quality randomized controlled trials examining the effects of tennis on BMD. The search covered literature from database inception to March 2025. Data were screened, extracted, coded, and statistically analyzed using Review Manager 5.3. Study selection was based on the PICOS criteria: (i) male tennis players compared to non-tennis players of the same age; (ii) tennis intervention group versus groups engaging in other regular physical activities; (iii) outcome indicators included bone mineral content (BMC) and BMD; and (iv) study design included cross-sectional studies, cohort studies, or controlled experiments.

**Result:**

A total of 10 articles involving 761 male participants aged between 10 and 26 were included in the analysis. Tennis intervention was found to significantly improve bone measures in several areas. Specifically, it led to increases in dominant arm BMC (SMD = 0.57 mg/mm, 95% CI [0.01, 1.13], P = 0.04), lumbar spine BMD (MD = 0.10 g/cm^2^, 95% CI [0.08, 0.11], P < 0.00001), dominant arm and radius BMD (SMD = 1.34 g/cm^2^, 95% CI [0.49, 2.80], P = 0.002), and right femur BMD (MD = 0.10 g/cm^2^, 95% CI [0.02, 0.18], P = 0.02). These differences were statistically significant (P < 0.05). Conversely, tennis had no significant effect on whole-body BMC (MD = 25.51 mg/mm, 95% CI [-38.43, 85.35], P = 0.43), non-dominant arm BMC (SMD = 0.03 mg/mm, 95% CI [-0.31, 0.37], P = 0.88), total body BMD (MD = 0.00 g/cm^2^, 95% CI [-0.01, 0.01], P = 0.97), femoral neck BMD (MD = 0.01 g/cm^2^, 95% CI [-0.00, 0.01], P = 0.30), or left femur BMD (MD = -0.01 g/cm^2^, 95% CI [-0.07, 0.05], P = 0.84). These findings were not statistically significant (P > 0.05). Additionally, subgroup analyses further revealed that tennis training exerted a targeted impact on BMD. Training durations of more than 7 years and at least 15 hours per week significantly improved BMD in the dominant arm and radius. Additionally, significant improvements in the non-dominant arm and radius BMD were observed in participants with over 2 years of training experience. Enhancement of greater trochanter BMD was closely associated with age of training initiation, with significant improvements noted when training began after age 15. In contrast, tennis had minimal influence on femoral neck BMD.

**Conclusion:**

Tennis effectively enhances BMD in the dominant arm, radius, lumbar spine, greater trochanter, and right lower limb. The effects are asymmetrical, favoring the dominant side. Therefore, to maintain balanced development of BMC and BMD in both limbs, it is recommended to include contralateral (non-dominant side) training during tennis practice.

**Systematic review registration:**

https://www.crd.york.ac.uk/PROSPERO/identifier: CRD42023486547. Registration date: March 10, 2025.

## Introduction

Bone mineral density (BMD)is a critical indicator of bone mass and a key predictor of osteoporosis, playing a vital role in overall human health. A reduction in bone mass significantly increases the risk of osteoporosis and fractures [1–6]. Osteoporotic fractures most commonly occur in the hip and spine; however, they can also affect the proximal humerus, wrist, ribs, ilium, ischiopubic branches, ankle, and other regions due to bone fragility. The burden of osteoporosis is substantial, as fragility fractures of all types can lead to serious complications, including death [7–10]. Moreover, the economic impact of osteoporosis is considerable, placing a heavy strain on healthcare systems globally [11].

Historically, osteoporosis was considered a disease of old age, primarily affecting individuals over the age of 60. However, in recent years, changing lifestyles have led to an increasing incidence of osteoporosis among younger populations [12]. One study found that although none of the students had developed osteoporosis, 32.1% of male students and 9.1% of female students already showed signs of low bone density and bone loss [13]. A related survey reported that the prevalence of low bone mass in individuals aged 40–49 reached 32.9%, with men accounting for 34.5% and women 31.4% [14]. Men also tend to be taller than women, and men who experience fractures have a higher risk of fatal complications. A prospective cohort study tracking fracture incidence and mortality over 10 years in individuals aged over 60 reported 952 low-trauma fractures and 461 deaths (48.4%) in women, compared to 343 fractures and 197 deaths (57.4%) in men [15]. Other studies have shown that men have higher mortality rates from hip and vertebral fractures than women [16–20]. For example, a 5-year prospective cohort study by Center et al. in Dubbo, Australia, found that mortality from femoral fractures was significantly higher in men than in women [21]. These findings highlight the urgent need for effective prevention and treatment strategies for male osteoporosis, which has become a growing focus in academic research.

Recent studies suggest that maximizing peak bone mass during adolescence and young adulthood is one of the most effective ways to prevent osteoporosis later in life [22]. During the growth period, adolescents’ bones exhibit significantly greater adaptability to mechanical loading compared to adults [23–25]. For more than two decades, physical activity and sports have been shown to improve BMD in children, adolescents, and adults alike [26–27]. Cross-sectional studies of adolescent athletes consistently demonstrate more significant BMD improvements than longitudinal studies of sedentary adults, indicating the strong impact of early physical activity on bone development [28–33].

Tennis is one of the most popular sports worldwide. It involves dynamic, full-body movements requiring quick changes in direction and a unilateral grip, creating an asymmetrical force distribution. These movement patterns expose various body parts to differing mechanical stimuli, influencing regional bone mass [34]. Repeated use of a specific limb over time leads to morphological adaptations, including increased bone and lean mass in the dominant side [35,36]. This asymmetrical loading model has garnered considerable interest among researchers studying the relationship between tennis and BMD. Numerous studies confirm that tennis has a beneficial effect on bone density in young individuals [37,38]. According to Wolff’s Law, bone adapts to mechanical loads through mechanotransduction, whereby sustained unilateral loading in tennis increases bone mass and volume on the dominant limb, resulting in interlimb asymmetry [39–41].

However, current research on the relationship between tennis and male BMD primarily focuses on the upper limbs. For instance, Laurent Chapelle et al. found that gender plays a role in upper limb bone mineral content (BMC) asymmetry in tennis players, with greater effects observed in males than females [42]. There is still a lack of comprehensive research on the impact of tennis on total and lower-body BMD in males. Therefore, this study aims to systematically evaluate the effects of tennis on male BMD across multiple body regions through meta-analysis. It also explores how training experience, frequency, and age influence male BMD.

## Methods

The systematic review and data extraction in this study were conducted in strict accordance with the Preferred Reporting Items for Systematic Reviews and Meta-Analyses (PRISMA) guidelines [43]. The review protocol was registered on March 10, 2025, and is available in the PROSPERO database (Registration ID: CRD42023486547).

### Literature search

This study strictly followed the PRISMA (Preferred Reporting Items for Systematic Reviews and Meta-Analyses) guidelines (Fig 1). All studies examining the impact of tennis on BMD published before March 2025 were retrieved from CNKI, Web of Science, Embase, PubMed, and other relevant databases. The search terms included combinations of the following keywords: “tennis” or “racket sports”; “male,” “male tennis player,” or “tennis player”; “cross-sectional,” “controlled experiment,” or “cohort studies”; and “BMD” or “BMC.” A combination of keywords and free-text terms was used in the search strategy, and no language restrictions were applied. Detailed search strategies for CNKI, Web of Science, Embase, and PubMed are provided in Table 1.

**Table 1:** PubMed retrieval strategy.

#1 (“Bone mineral density”(All Fields) OR “Bone mineral content”(All Fields) OR “Bone mass”(All Fields) OR “Bone composition”(All Fields) OR “Osteoporosis”(All Fields)) AND “Body composition”(MeSH Terms)
#2“Bone mineral density”(Title/Abstract) OR “Bone mineral content”(Title/Abstract) OR “Bone mass” (Title/Abstract) OR “Bone composition”(Title/Abstract) OR “Body composition” (Title/Abstract)
#3 #1 OR #2
#4 “Body composition”(Title/Abstract) OR “Bone mineral density”(Title/Abstract) OR “Bone mineral content” (Title/Abstract) OR “Osteoporosis” (Title/Abstract) OR “Bone mass” (Title/Abstract)
#5“Teenager” (Title/Abstract) OR “College athlete” (Title/Abstract) OR “Male tennis players” (Title/Abstract) OR“College tennis players”(Title/Abstract)
#6“Body exercise” (Title/Abstract) OR “Tennis” (Title/Abstract) OR “Racket sports”(Title/Abstract) OR “Tennis”(Title/Abstract)
#7 “Controlled experiment” (Publication Type) OR “Cross-sectional” (Publication Type) OR “cohort studies” (Publication Type)
#8 #3 AND #4 AND #5 AND #6 AND #7

**Fig 1:**
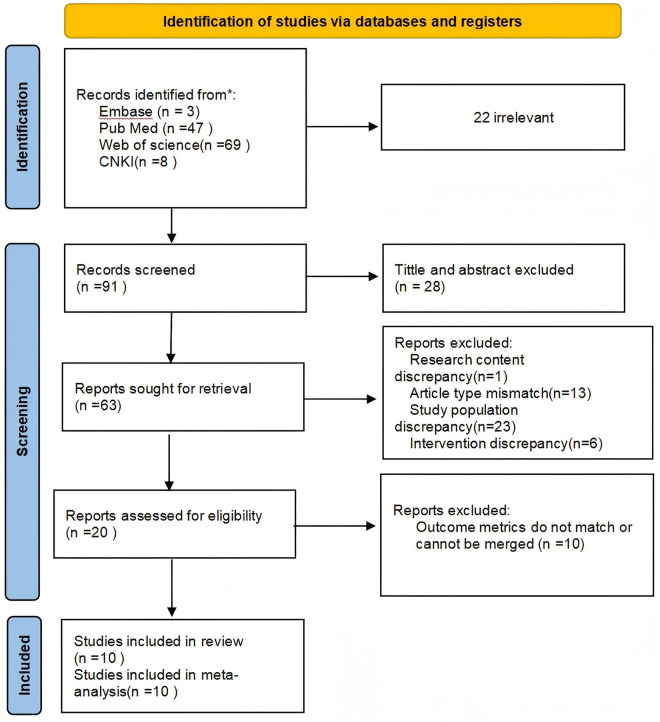
PRISMA selection process summary.

### Inclusion and exclusion criteria

The inclusion criteria were as follows: (1) the research subjects were male tennis players and non-tennis players of the same age; (2) comparative studies assessing outcomes before and after the intervention; (3) studies involving tennis intervention alone or in combination with other interventions; and (4) research designs limited to cross-sectional studies, cohort studies, or randomized controlled trials.

The exclusion criteria were as follows: (1) the research subjects are female tennis players or people with other underlying diseases; (2) studies where the control group did not consist of participants engaged in regular physical activity or other sports; (3) studies lacking BMC and BMD as outcome indicators; (4) research that did not follow a cross-sectional, cohort, or controlled experimental design; and (5) studies where data is incomplete or valid data cannot be extracted, such as conference presentations, case studies, extraction analyses, design schemes, reviews, or editorial articles.

Additionally, the PICOS framework was used to guide the selection of relevant studies (Table 2). Only studies published in English or Chinese academic journals were included. This review is based on studies that meet the above criteria and focus on the effects of tennis on male BMD.

**Table 2:** PICOS-based eligibility criteria (participation, intervention, comparison, outcomes, and study design).

PICOS	Criteria
**Participation**	male tennis players – non-tennis players of the same age
**Intervention**	Tennis intervention
**Comparison**	Tennis exercise intervention group – Regular comparison of other physical activity groups
**Outcome**	Whole body BMC, dominant arm BMC, non-dominant arm BMC, whole body BMD, dominant arm radius BMD, non-dominant arm radius BMD, lumbar spine BMD, left femur BMD, right femur BMD, greater trochanter BMD, and femoral neck BMD
**Study design**	Cross-sectional or cohort studies or controlled experiment

BMC:Bone Mineral Content;BMD:Bone Mineral Density.

### Literature screening and quality assessment

Three researchers collaboratively conducted the literature screening, data extraction, and assessment of risk of bias and study quality. Initially, two researchers screened for duplicates and obviously irrelevant literature by reviewing the titles. They then further assessed the abstracts and full texts based on the predefined inclusion and exclusion criteria to determine eligibility. To ensure data completeness, if full-text articles were not accessible via databases, the researchers obtained them by searching the journals directly or contacting the corresponding authors via email.

After initial selection, the two researchers cross-checked the included studies for consistency. In cases of disagreement, a third researcher reviewed the studies to resolve any discrepancies and reach consensus. Ultimately, all three researchers jointly evaluated the risk of bias using the Joanna Briggs Institute (JBI) risk-of-bias assessment tools for cross-sectional and cohort studies.

Methodological quality was further assessed independently by two reviewers using the QualSyst tool [44], which consists of 14 criteria (e.g., “Question/objective clearly described”). Each item was scored as follows: 0 for “No,” 1 for “Partial,” and 2 for “Yes.” The total score for each study was then calculated to determine its quality level: scores <55% indicated low quality, 55–75% indicated moderate quality, and >75% indicated high quality. After individual evaluations, a third reviewer joined the two initial reviewers in a consensus meeting to reconcile any differences and finalize the quality ratings.

Among the included studies, only Guo Liang’s study explicitly described a method for random sequence generation. The remaining nine studies did not report this information. All ten studies had complete data, showed no evidence of selective reporting, and presented no other significant sources of bias. As the included studies were cross-sectional or cohort in design, blinding was not applied. The detailed quality evaluation results are presented in Table 3.

**Table 3:** “Qualsyst” of quality assessment.

Calbet et al 1998	Claudia 2008	Guo Liang 2010	Joaquin 2010	Tan Keli 2010	Yang Zhan Guang 2018	Li Feng Shuan 2019	Kontulainen 1999	Ashizawa et al. 1999	Haapasalo 2000	Publication
2	2	2	2	1	2	2	2	1	2	Question/objective described
2	2	1	2	1	2	2	1	2	2	Appropriate study design
2	1	2	1	2	2	2	1	1	2	Characteristic Sufficiently described
NA	NA	2	NA	NA	NA	NA	NA	NA	NA	Random allocation
0	0	0	0	0	0	0	0	0	0	Researchers blinded
0	0	0	0	0	0	0	0	0	0	Subjects blinded
1	2	1	1	2	2	2	1	1	2	Outcome measures well-defined and robust to bias
1	2	2	2	2	2	2	1	1	1	Appropriate sample size
2	2	2	2	2	2	2	2	2	2	Analytic methods well-described
2	2	2	1	1	2	2	2	2	1	Estimate of variance reported
0	1	1	0	0	0	1	0	0	0	Controlled for confounding
2	2	1	2	2	2	2	1	2	2	Results reported in detail
2	2	1	2	2	2	2	1	2	2	Conclusion supported by results?
Medium	High	Medium	Medium	Medium	Medium	High	Medium	Medium	Medium	Rating

NA = Not applicable.

### Data extraction and risk of bias assessment

The main contents of data extraction included general study information (author name, year of publication, and journal name), basic characteristics of study participants (age, gender, sample size), and details of the exercise intervention (duration, frequency, type, and intensity). Additionally, data relevant to risk of bias assessment, specific intervention measures, and outcome indicators with corresponding results were collected.

The risk of bias in the included studies was assessed using standardized tools developed by the JBI for evaluating cross-sectional and cohort studies [45]. For cross-sectional studies, the JBI quality appraisal tool consisted of eight criteria: clearly defined inclusion criteria, description of study participants and settings, measurement methods for exposure factors, assessment methods for health outcomes, identification of confounding factors, strategies to address these confounders, outcome measurement methods, and appropriateness of data analysis. Items 4 and 3 of the JBI critical appraisal tools for analytical cross-sectional and cohort studies, respectively, were considered not applicable, as all included studies utilized either DXA or pQCT scans for measuring BMC.

For cohort studies, the JBI checklist included eleven items: inclusion criteria, description of study subjects and settings, measurement of exposure factors, identification and control of confounding variables, outcome measurement methods, assessment of outcomes, duration and adequacy of follow-up, completeness of follow-up, management of incomplete data, and data analysis methods. Each item was evaluated as “Yes,” “No,” “Unclear,” or “Not applicable.” Final decisions regarding study inclusion, exclusion, or the need for additional information were reached through group discussion among the research team.

### Grouping standard

The grouping criteria for extraction and analysis were primarily based on the variables used in each study, which varied depending on the study objectives and the specific problems being addressed. These grouping factors included the characteristics of the interventions, treatment protocols, intervention conditions, frequencies, and other relevant variables that could influence the observed outcomes. To ensure the accuracy and validity of the subgroup analysis, random allocation of variables, excluding the subgroup-defining factors, was considered essential. This approach helps minimize intergroup differences caused by confounding variables and reduces the risk of bias in subgroup outcomes.

Following a review of the intervention characteristics in the 10 included studies, substantial differences were noted in terms of participant age, years of training, and training frequency. Consequently, three subgroup analyses were conducted:

1) Training duration subgroup: Participants were grouped based on differences in years of training. Those with similar ages and training frequencies but with approximately one year of experience were categorized into the “≈1 year” subgroup. Participants with longer training histories, characteristically more than 7 years, were classified into the “>7 years” subgroup.
2) Training frequency subgroup: In three studies where training frequency was a notable distinguishing factor (while other intervention features were vague or incomplete), subgrouping was based on weekly training hours. Participants training more than 5 hours per week were assigned to the “>5 h/w” group, and those training less than 5 hours per week were assigned to the “<5 h/w” group.
3) Age subgroup: Participants were divided according to age. Those older than 15 years were categorized into the “>15” group, while those 15 years old or younger were placed in the “<15” group.

These subgroup analyses were designed to explore how training experience, frequency, and age influence the effects of tennis on BMD.

### Statistical analysis and meta-analysis

Data analysis was conducted using RevMan 5.3 software. Since the outcome indicators reported in the included studies were continuous variables and measured using different methods, two approaches were applied: when the units of measurement were consistent across studies, the effect size was expressed as a mean difference (MD); when the measurement units differed, the effect size was reported as a standardized mean difference (SMD). All effect sizes were presented with 95% confidence intervals (CIs).

In this study, SMD was used to calculate the effect size for four indicators, dominant arm BMC, non-dominant arm BMC, dominant radial arm BMD, and non-dominant radial arm BMD, due to the use of two different measuring instruments. All other indicators were analyzed using MD.

Heterogeneity among studies was assessed using the I^2^ statistic, with a threshold of I^2^ < 50% and P < 0.05 indicating low heterogeneity. If heterogeneity was low, a fixed-effects model was applied. For analyses with high heterogeneity (I^2^ ≥ 75%), subgroup analyses were conducted to explore potential confounding variables influencing the effect of tennis on male BMD. All meta-analyses were performed using Comprehensive Meta-Analysis software (version 2; Biostat Inc., Englewood, NJ, USA) [46,47].

**Fig 2:**
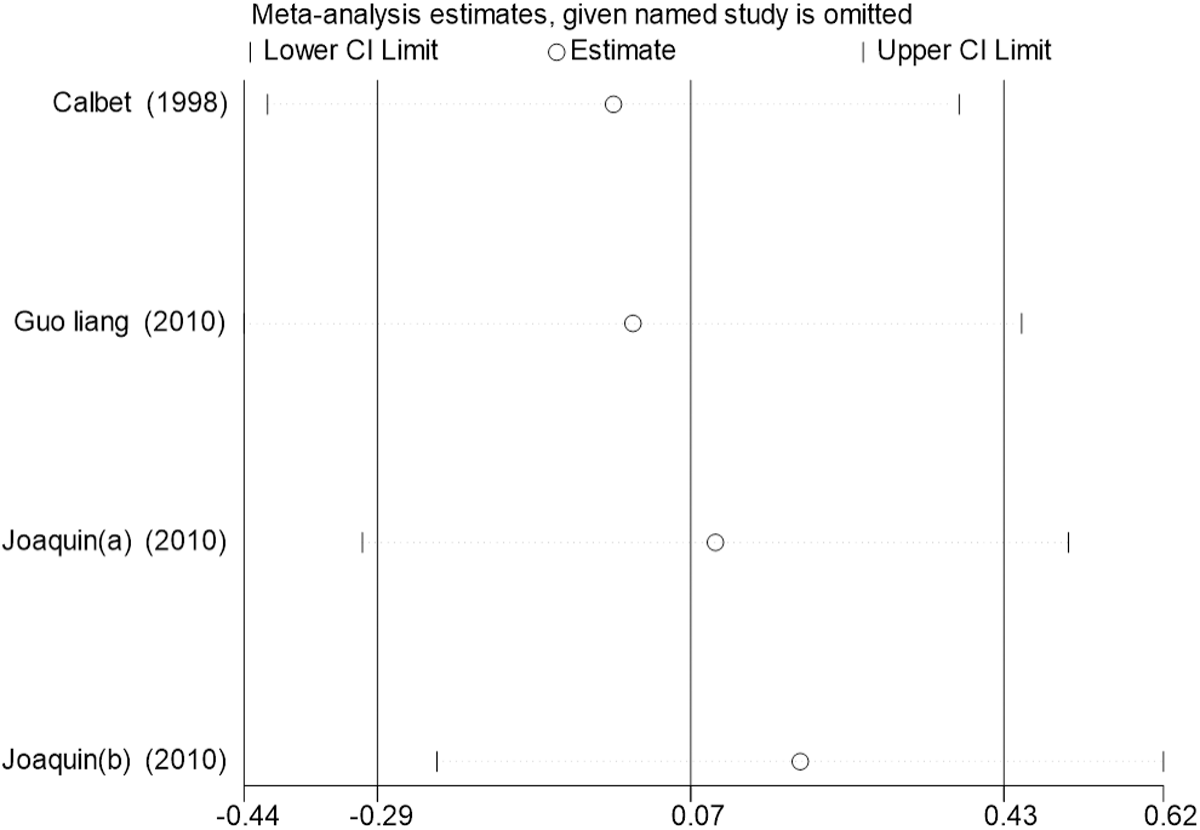
Sensitivity analysis diagram of the impact of tennis on whole body BMC in male.

Sensitivity analysis for the 11 outcome indicators, including whole-body BMC, whole-body BMD, dominant arm BMC, non-dominant arm BMC, dominant radial arm BMD, non-dominant radial arm BMD, lumbar spine BMD, greater trochanter BMD, femoral neck BMD, left femur BMD, and right femur BMD, was performed using Stata 18.0. The results showed that the inclusion or exclusion of any individual study had a negligible impact on the overall effect size, and the estimated effect size of each study fell within the confidence interval range. This indicates that the results are robust and reliable (Figs 2).

**Fig 3:**
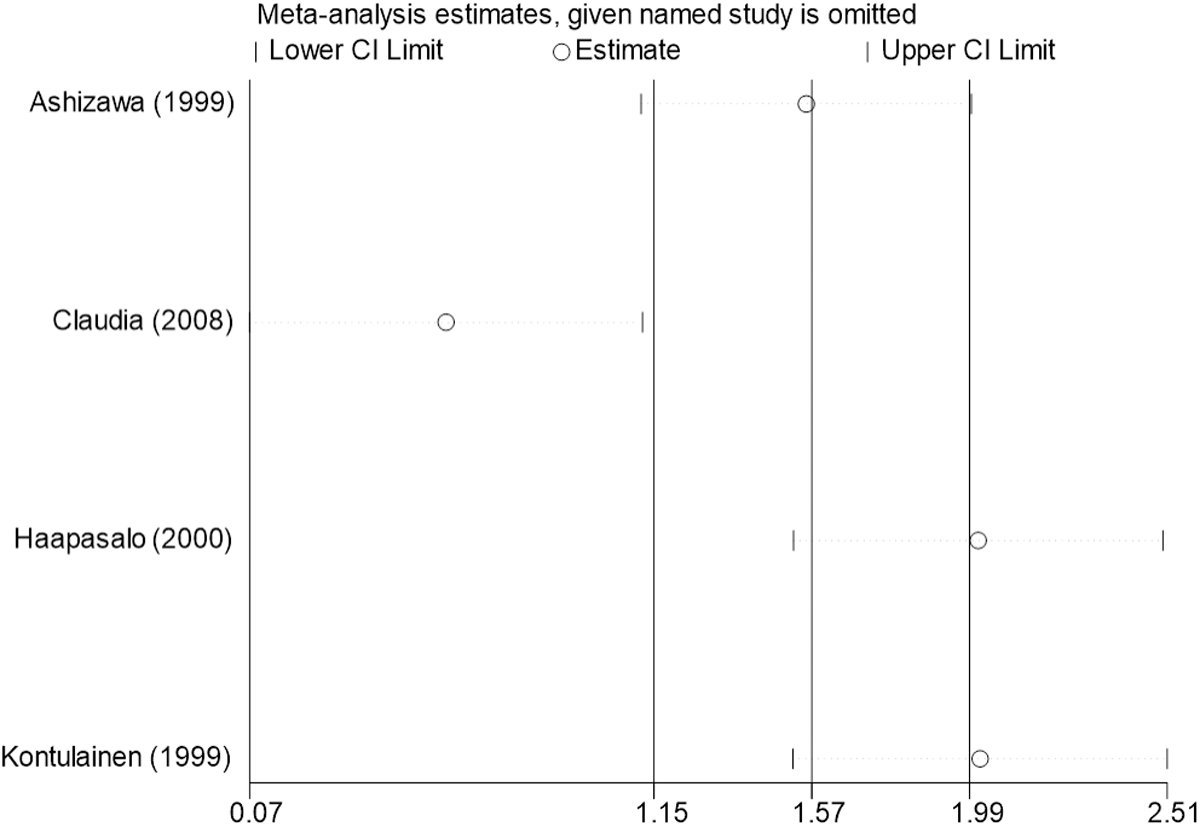
Sensitivity analysis diagram of the impact of tennis on whole-body BMD in male.

**Fig 4:**
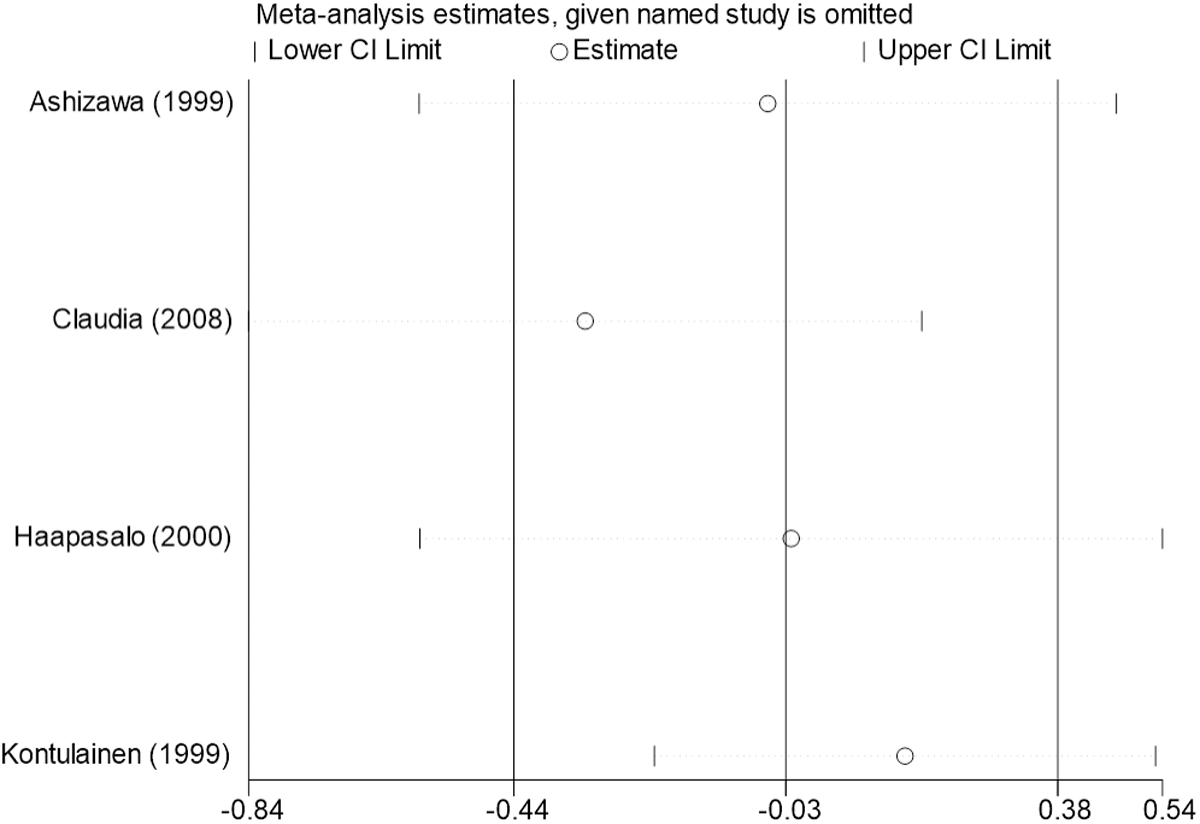
Sensitivity analysis diagram of the impact of tennis on dominant arm BMC in male.

**Fig 5:**
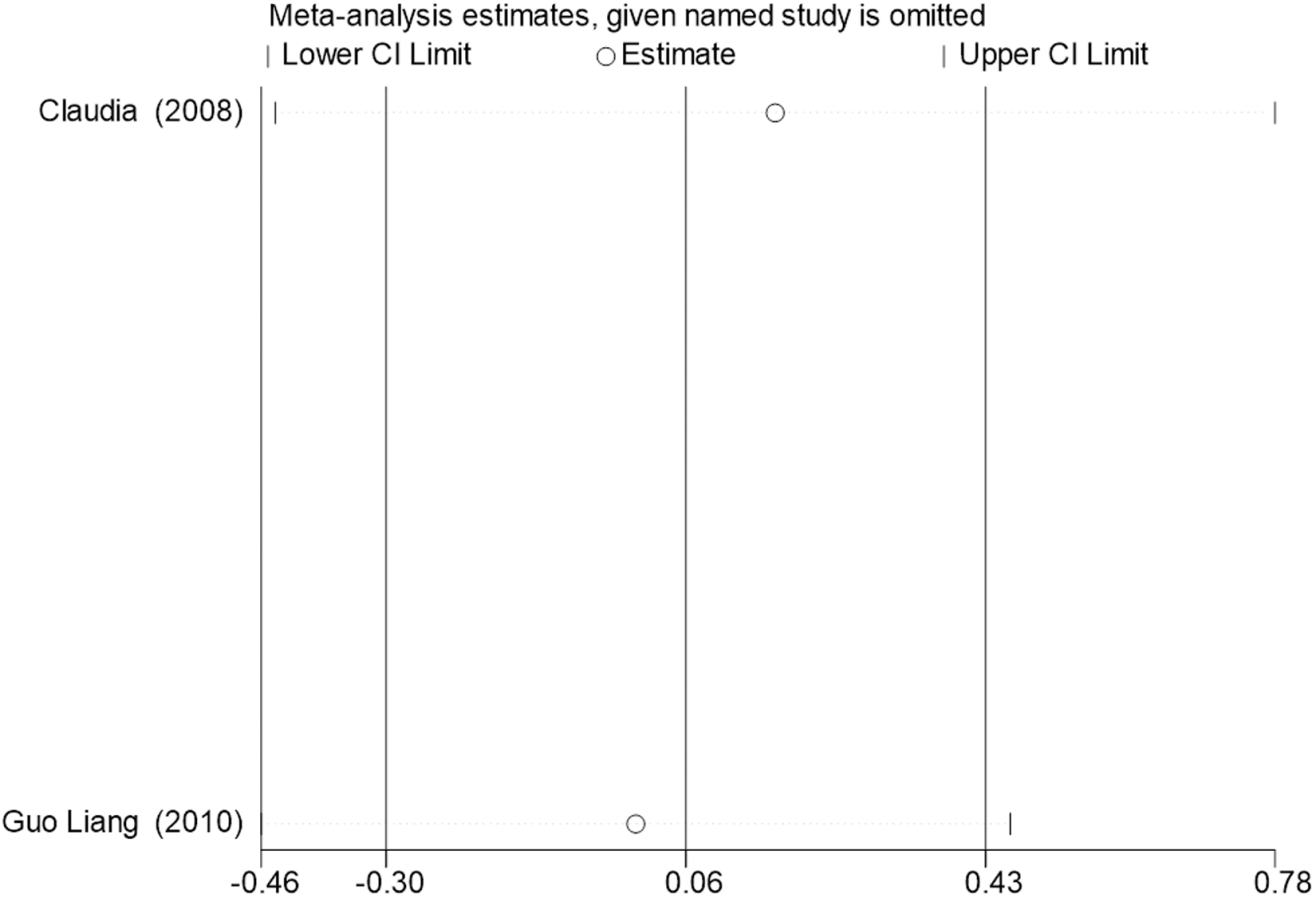
Sensitivity analysis diagram of the impact of tennis on non-dominant arm BMC in male.

**Fig 6:**
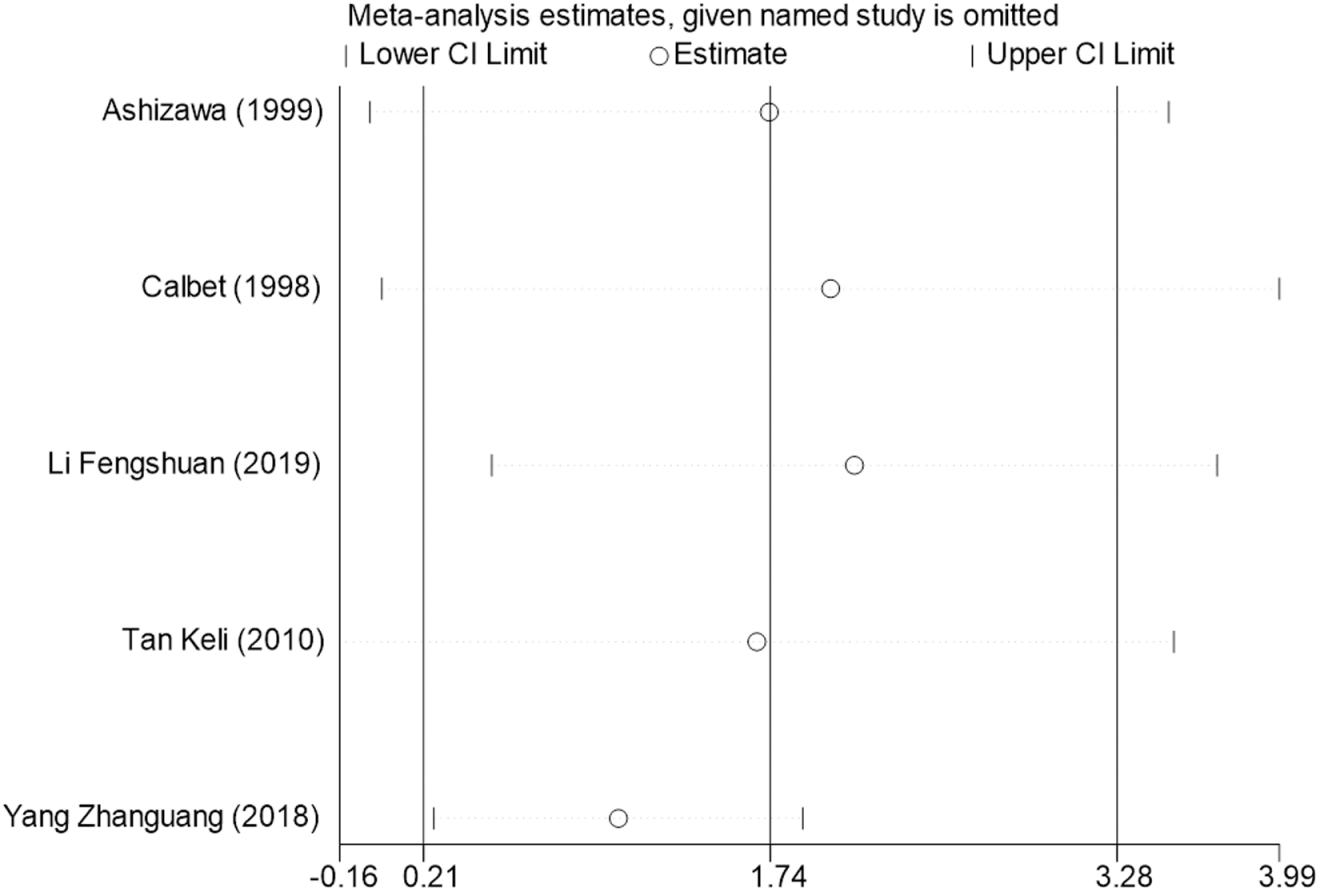
Sensitivity analysis diagram of the impact of tennis on dominant radial arm BMD in male.

**Fig 7:**
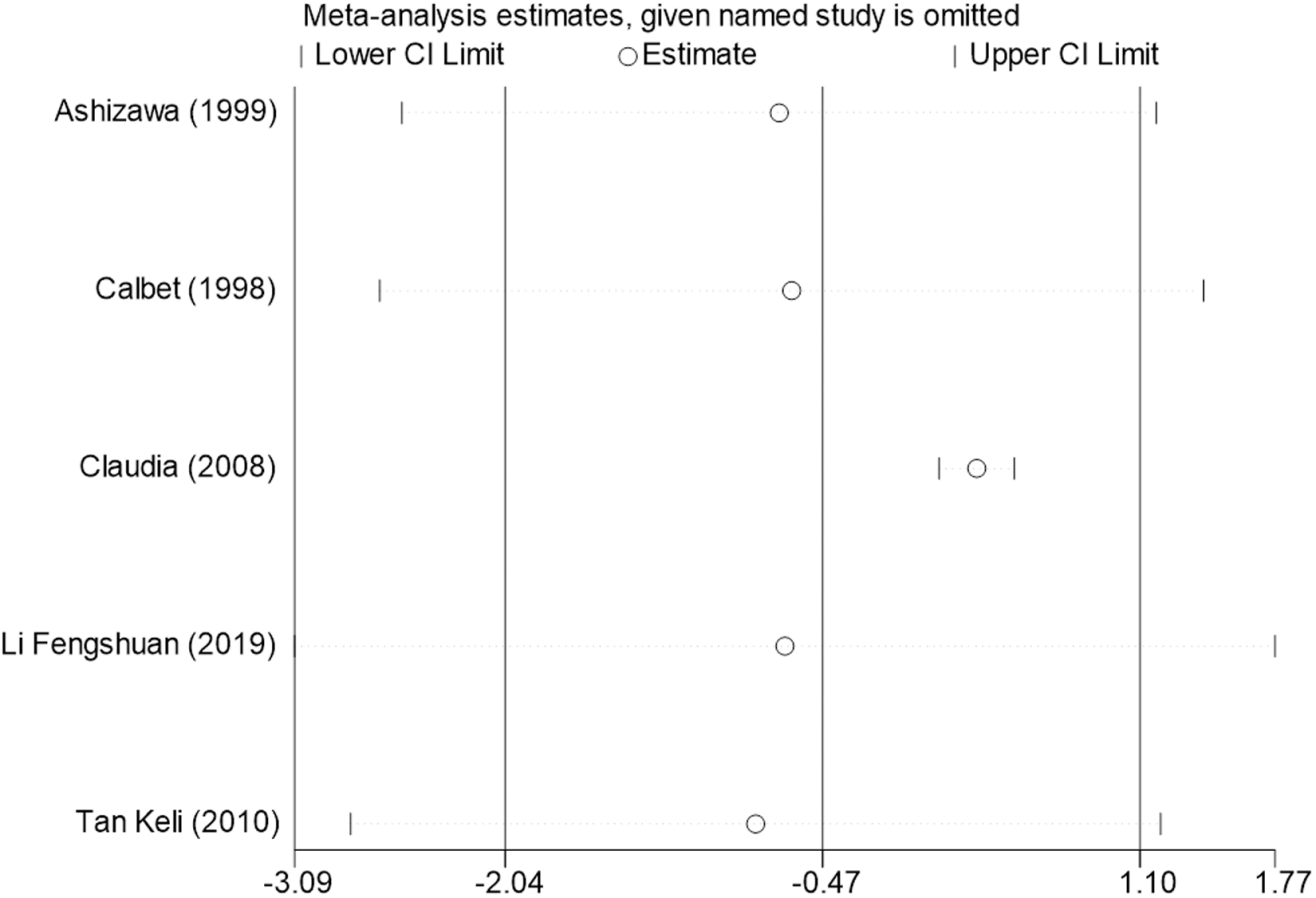
Sensitivity analysis diagram of the impact of tennis on non-dominant radial arm BMD in male.

**Fig 8:**
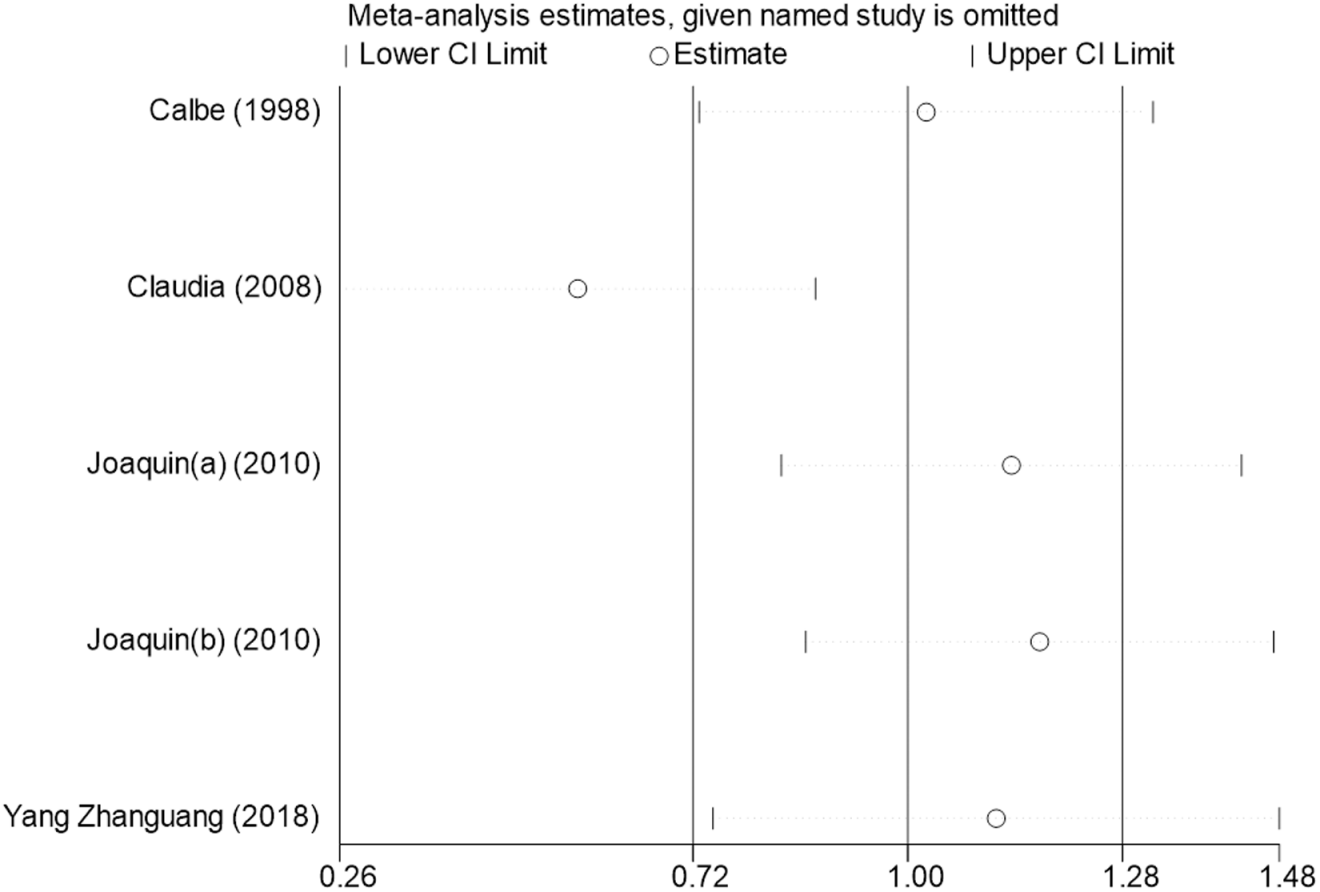
Sensitivity analysis diagram of the impact of tennis on lumbar spine BMD in male.

**Fig 9:**
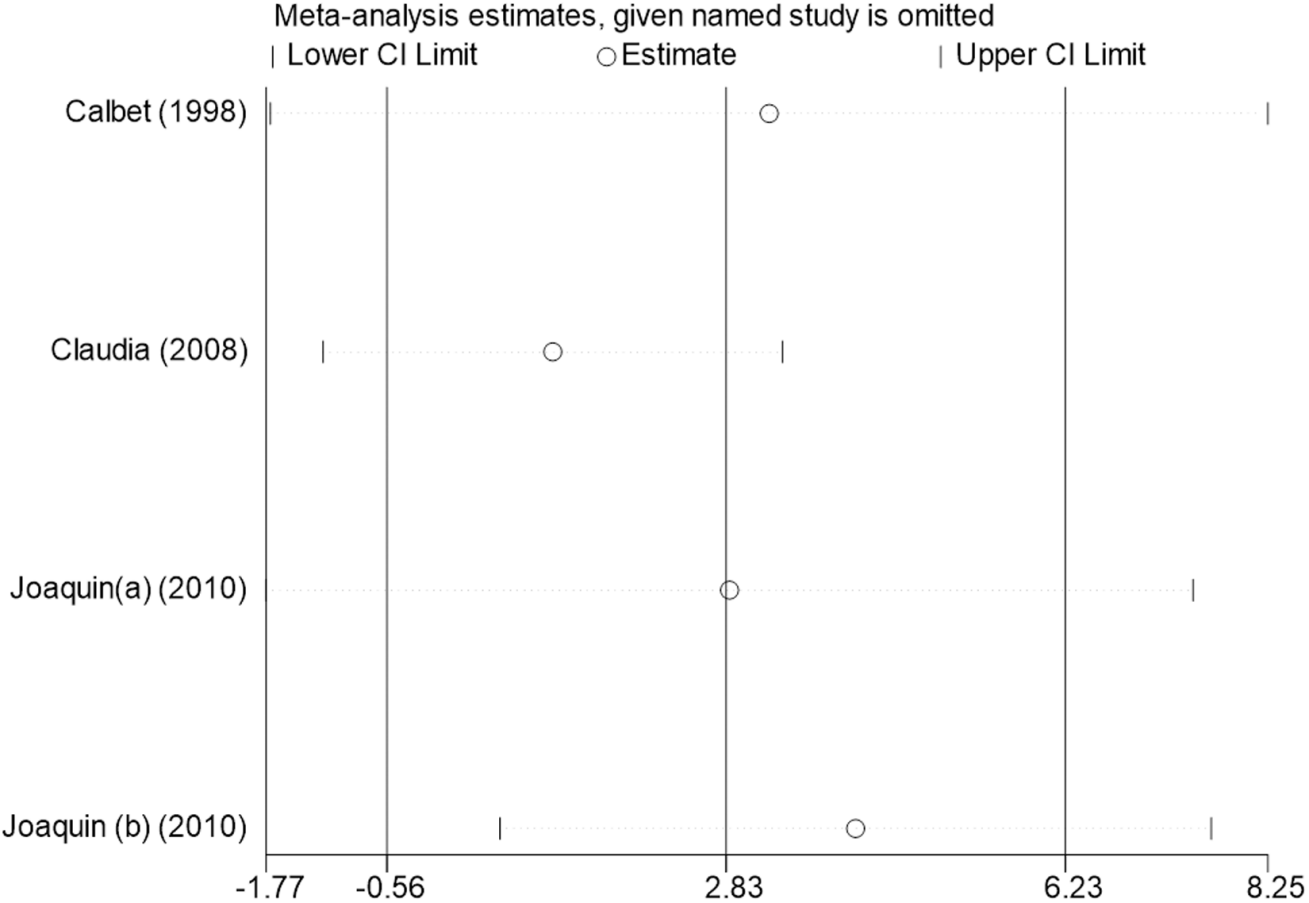
Sensitivity analysis diagram of the impact of tennis on greater trochanter BMD in male.

**Fig 10:**
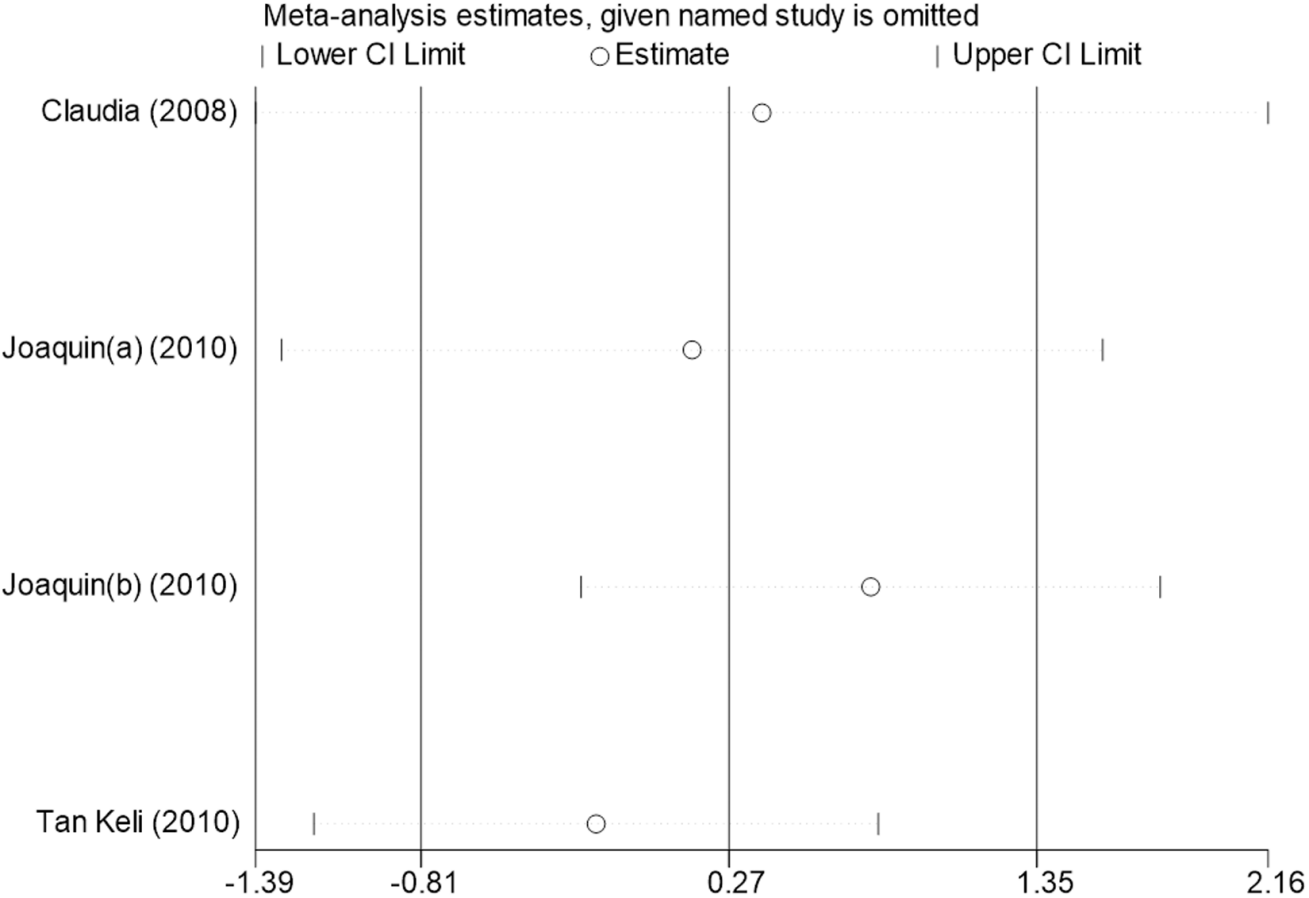
Sensitivity analysis diagram of the impact of tennis on femoral neck BMD in male.

**Fig 11:**
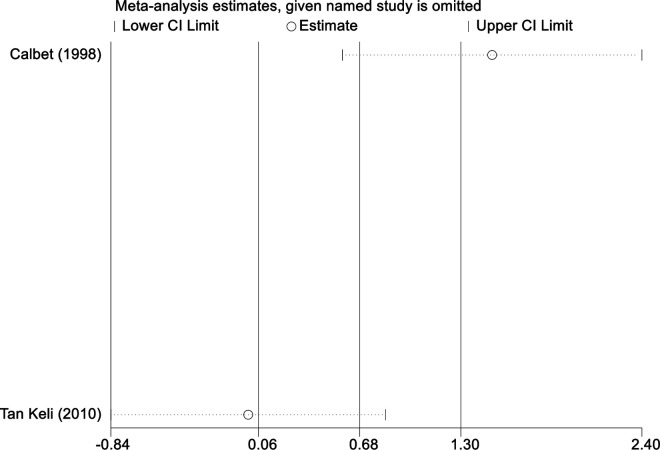
Sensitivity analysis diagram of the impact of tennis on left femur BMD in male.

**Fig 12:**
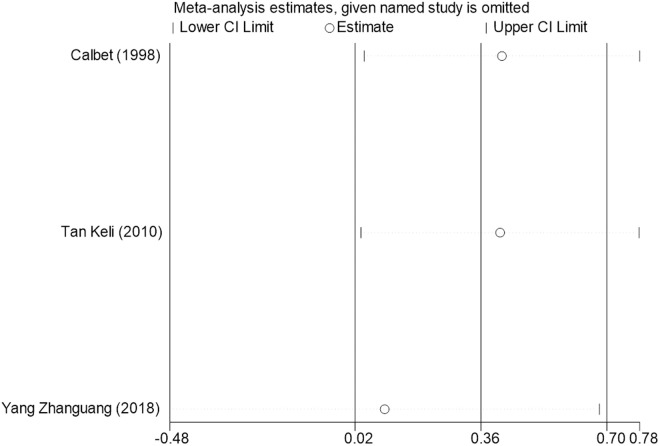
Sensitivity analysis diagram of the impact of tennis on right femur BMD in male.

Publication bias was assessed using funnel plots along with Egger’s test [48] and Begg’s test [49]. The funnel plots () showed a generally symmetrical distribution of studies, suggesting minimal publication bias. Results of Egger’s and Begg’s tests for each outcome indicator were as follows:

**Fig 13:**
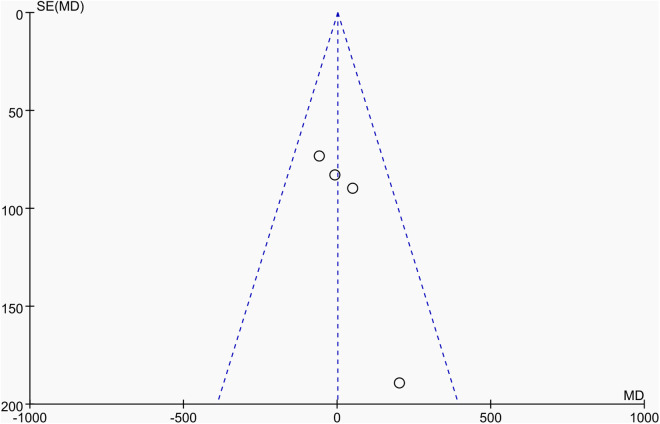
Funnel plot of the effect of tennis on whole-body BMC in male.

**Fig 14:**
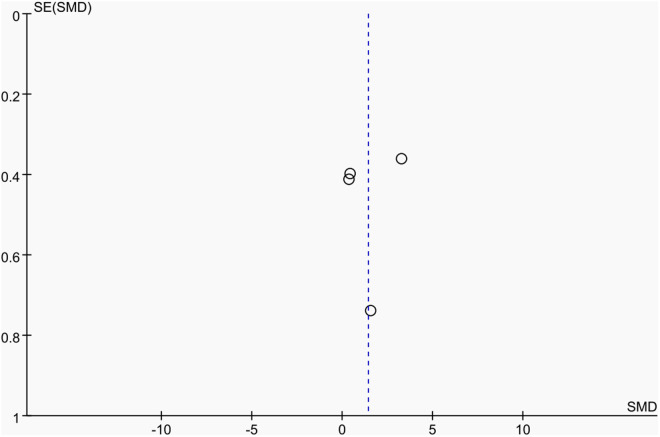
Funnel plot of the effect of tennis on whole-body BMD in male.

**Fig 15:**
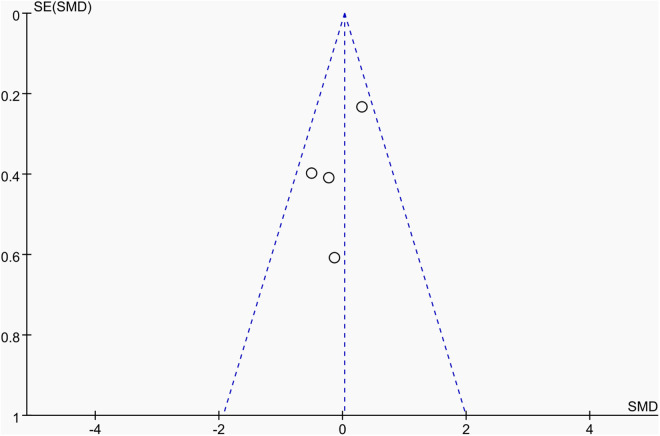
Funnel plot of the effect of tennis on dominant arm BMC in male.

**Fig 16:**
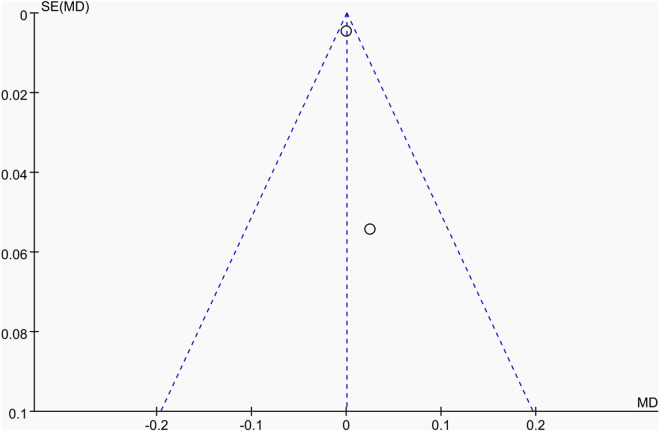
Funnel plot of the effect of tennis on non-dominant arm BMC in male.

**Fig 17:**
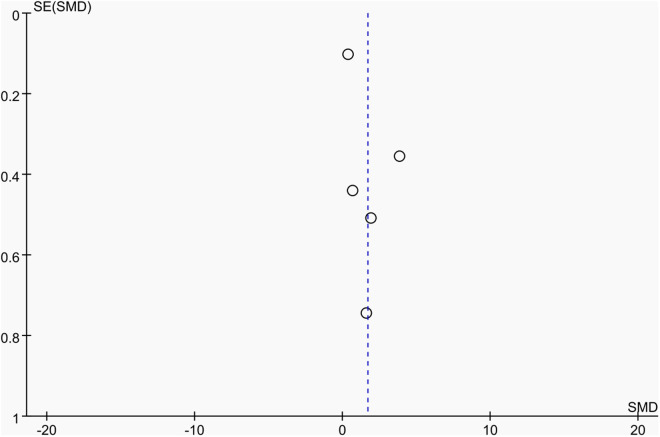
Funnel plot of the effect of tennis on dominant radial arm BMD in male.

**Fig 18:**
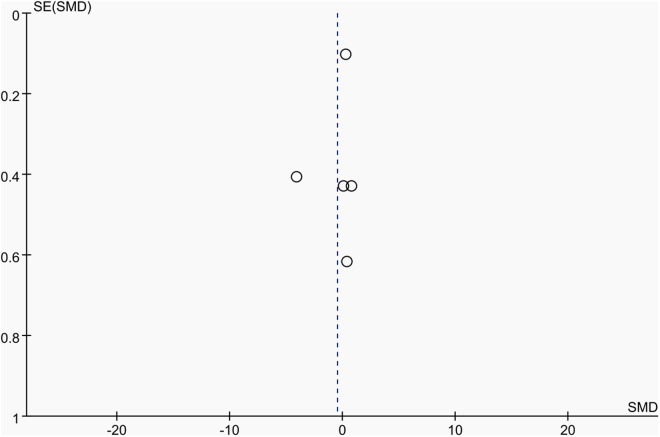
Funnel plot of the effect of tennis on non-dominant radial arm BMD in male.

**Fig 19:**
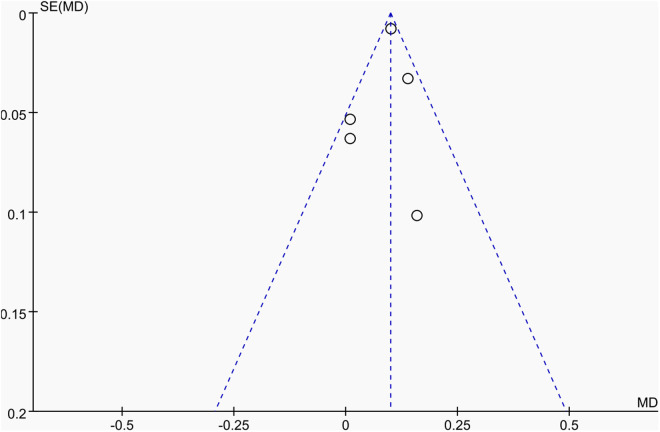
Funnel plot of the effect of tennis on lumbar spine BMD in male.

**Fig 20:**
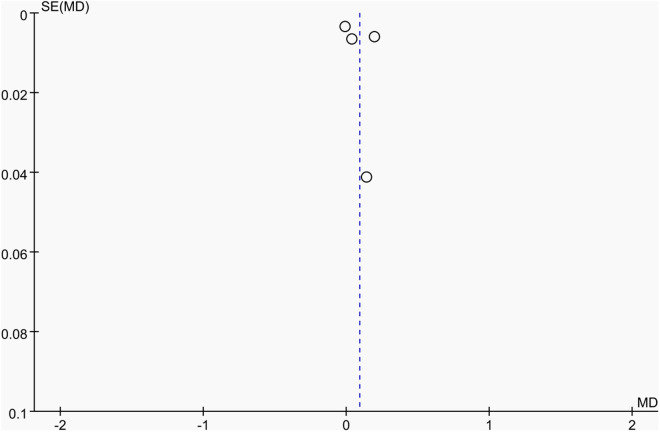
Funnel plot of the effect of tennis on greater trochanter BMD in male.

**Fig 21:**
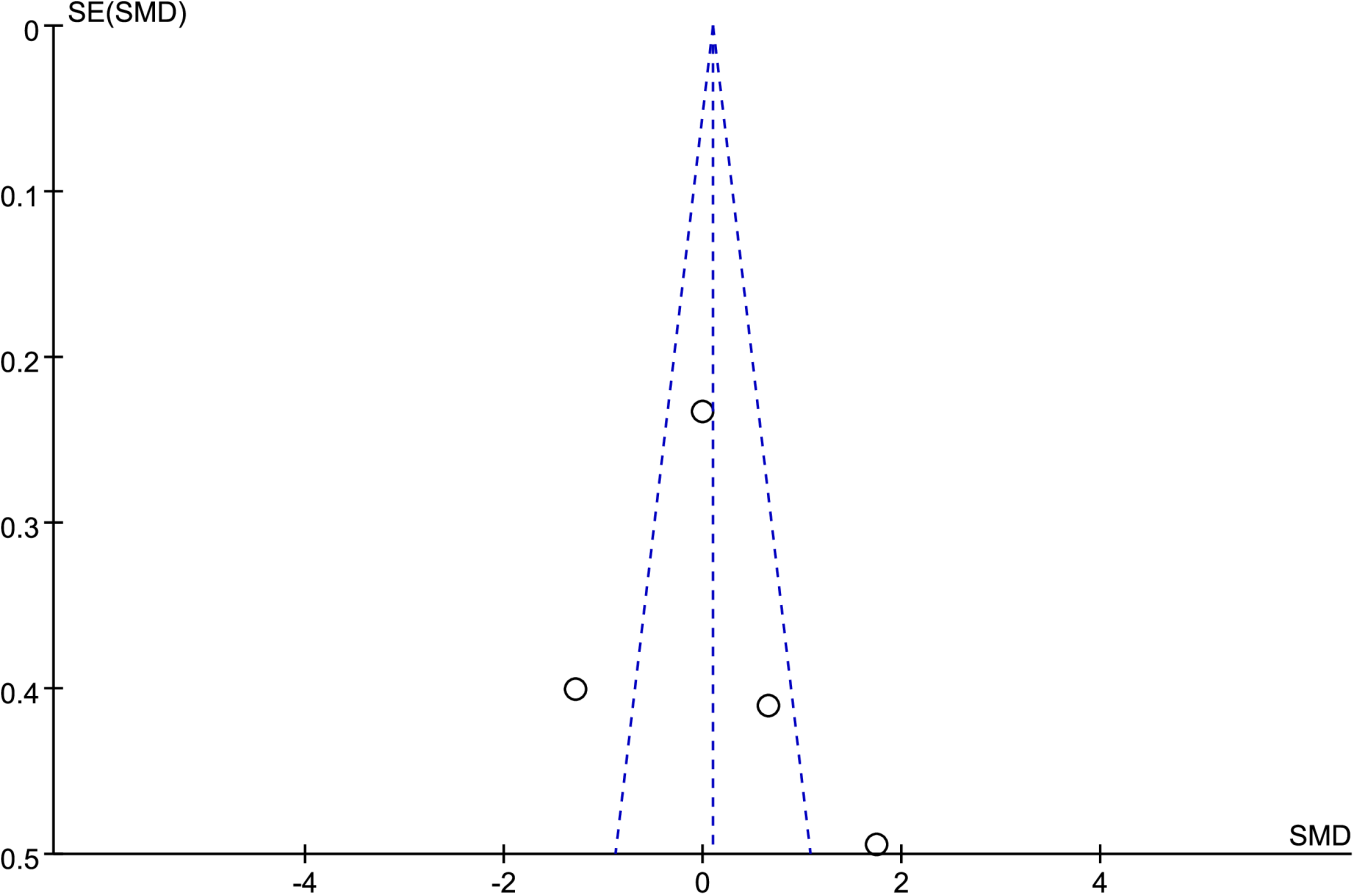
Funnel plot of the effect of tennis on femoral neck BMD in male.

**Fig 22:**
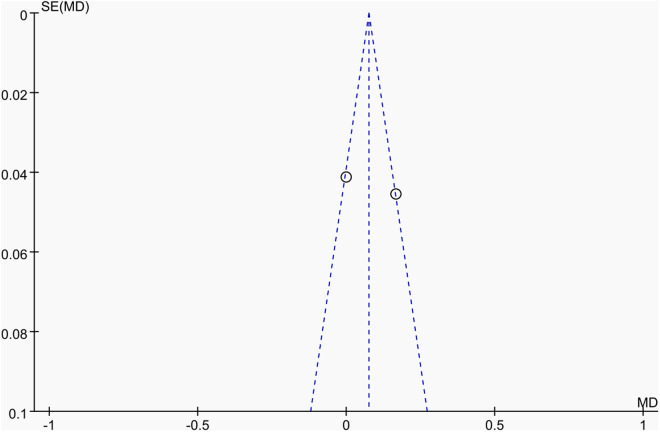
Funnel plot of the effect of tennis on left femur BMD in male.

**Fig 23:**
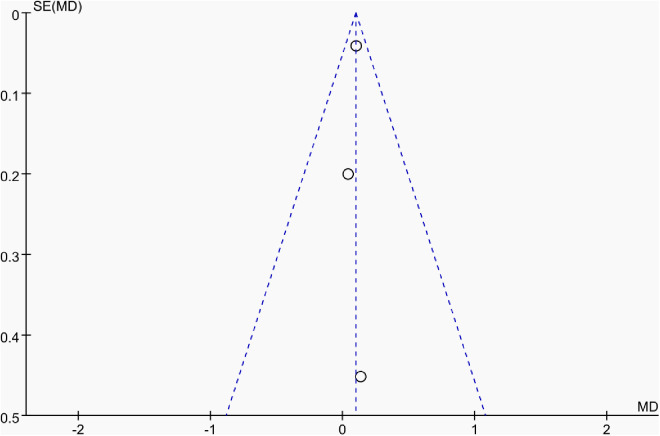
Funnel plot of the effect of tennis on right femur BMD in male.

Results of Egger’s and Begg’s tests for each outcome indicator were as follows: For whole-body BMC, the P-values were 0.8010 (Egger) and 0.7341 (Begg). For dominant arm BMC, the values were 0.0171 (Egger) and 1.000 (Begg), while for non-dominant arm BMC, they were 0.2671 and 1.000, respectively. Dominant radial arm BMD showed P-values of 0.058 (Egger) and 0.8065 (Begg), and non-dominant radial arm BMD had values of 0.542 and 0.8065. Lumbar spine BMD showed P-values of 0.227 (Egger) and 0.8065 (Begg). For greater trochanter BMD, the values were 0.011 (Egger) and 0.0894 (Begg). Femoral neck BMD yielded P-values of 0.676 (Egger) and 0.3082 (Begg), while left femur BMD had the same Egger value (0.676) and a Begg value of 0.2963. Finally, right femur BMD showed P-values of 0.493 (Egger) and 0.2963 (Begg).

With the exception of dominant arm BMC, all P values were greater than 0.05, indicating no significant publication bias. While Egger’s test for dominant arm BMC yielded P < 0.05, Begg’s test did not, and the funnel plot suggested only a minor degree of bias. Additionally, due to the small sample size of whole-body BMD data, Egger’s and Begg’s tests could not be conducted for this outcome. Nevertheless, the funnel plot for whole-body BMD showed a relatively symmetrical shape, indicating a low likelihood of publication bias.

In summary, across the 11 outcome indicators, the results collectively suggest that publication bias was minimal or absent.

## Result

A total of 113 studies related to the effects of tennis on BMD were retrieved. After screening based on the inclusion and exclusion criteria [37,38,50–57], 10 studies were selected for analysis, involving a total of 761 participants. The literature screening process is illustrated in Fig 1, and the basic characteristics of the included studies are presented in Tables 4 and 5.

**Table 4:** Includes the basic information of the literature.

Study	Sample size (T/C)	Chronological age (yrs)	BMI	Starting age (yrs)	Playing experience (yrs)	Training volume (hrs/ wk)	Nation	Research type	Primary outcome measure
**DAX**									
Calbet et al. 1998 [37]	9/14	26.2 ± 5.6 24.2 ± 2.8	23.86 ± 4.73 23.64 ± 2.4	NR	17 ± 6	25 ± 8	Spain	Cross section	(1)(5)(6)(7)(8)(10)(11)
Claudia 2008 [38]	44/32	15 ± 0.3 15 ± 0.4	20.5 ± 0.3 21.4 ± 0.6	NR	>1	>5	Brazil	Cross section	(2)(3)(4)(6)(7)(8)(9)
Guo Liang 2010 [44]	16/30	13.8 ± 1.84 13.8 ± 1.42	19.29 ± 0.98 19.72 ± 0.64	NR	>3	NR	China	Cross section	(1)(4)
Joaquin 2010 [45]	10/14/17	10.8 ± 0.7 10.4 ± 1.1 10.5 ± 0.7	17.46 ± 0.82 17.58 ± 0.67 18.89 ± 0.69	6.3 ± 1.9 6.8 ± 2.2	4.9 ± 2.2 3.5 ± 1.8	10.8 ± 2.3 3.1 ± 1.2	Spain	Cross section	(1)(7)(8)(9)
Tan Keli 2010 [46]	15/15	21.07 ± 1.87 21.38 ± 1.69	21.57 ± 0.97 22.64 ± 1.39	NR	7.7 ± 1.12	NR	China	Cross section	(5)(6)(9)(10) (11)
Yang Zhanguang 2018 [47]	36/60	NR	NR	NR	>1	>5	China	Cross section	(5)(7)(11)
Li Fengshuan 2019 [50]	188/200	13-17	19.74 ± 2.07 19.51 ± 2.16	NR	>2	NR	China	Cross section	(5)(6)
Kontulainen 1999 [53]	13/13	26.0 ± 5.1 26.2 ± 5.9	22.1 ± 3.1 23.5 ± 1.8	11	15.5 ± 5.1	7.6 ± 7.2	Finland	Cross section	(2)(3)
**pQCT**									
Ashizawa et al. 1999 [51]	6/5	18-24	21.61 ± 0.65 20.63 ± 1.3	12.8 ± 1.5	7.3 ± 1.1	2.5 ± 4	NR	Cross section	(2)(3)(5)(6)
Haapasalo 2000 [52]	12/12	19.20 ± 0.61 23.31 ± 0.94	29.4 ± 4.8 29.4 ± 5.3	10 ± 3	>19	2.7 ± 1.8	Finland	Cohort study	(2)(3)

DAX:Dual X-Ray Absorptiometry; pQCT:Peripheral Quantitative Computed Tomography;

1) whole BMC; 2) dominant arm BMC; 3) non-dominant arm BMC; 4) whole BMD; 5) Dominant radial arm BMD; 6) non-dominant radial arm BMD; 7) lumbar spine BMD; 8) trochanter greater BMD; 9) femoral neck BMD; 10) Left femur BMD; 11) Right femur BMD.

**Table 5:** Specific values of included research indicator.

Study	index										
	BMC(Bone Mineral Content) (mg/mm)			BMD(Bone Mineral Density) (g/cm^2^)							
	Whole body (M ± SD) (T/C/P)	dominant arm (M ± SD) (T/C)	non-dominant arm (M ± SD) (T/C)	whole body (M ± SD) (T/C)	lumbar spine (M ± SD) (T/C)	dominant arm and radius (M ± SD) (T/C)	non-dominant arm and radius (M ± SD) (T/c)	greater trochanter (M ± SD) (T/C)	femoral neck (M ± SD) (T/C)	left femur (M ± SD) (T/C)	right femur (M ± SD) (T/C)
Calbet 1998	3078 ± 476				1.25 ± 0.29	0.874 ± 0.5	0.821 ± 0.083		0.94 ± 0.11	1.426 ± 0.102	1.439 ± 0.101
	2876 ± 383				1.09 ± 0.12	0.821 ± 0.087	0.808 ± 0.088		0.80 ± 0.07	1.426 ± 0.087	1.398 ± 0.74
	NR				P = 0.09	P < 0.001	NR		p < 0.0001	NR	NR
Claudia 2008		173.7 ± 7.4	143.0 ± 7.5	1.1 ± 0.02	1.1 ± 0.03		0.7 ± 0.02	1.0 ± 0.02	1.1 ± 0.03		
		146.5 ± 9.1	140.4 ± 9.3	1.1 ± 0.02	1.0 ± 0.04		0.8 ± 0.03	0.9 ± 0.03	1.1 ± 0.03		
		p = 0.023	P = 0.827	P = 0.602	P = 0.198		P = 0.028	P = 0.032	P = 0.068		
Guo Liang 2010	1820.76 ± 318.95			0.995 ± 0.201			0.995 ± 0.201				
	1769.10 ± 226.56			0.970 ± 0.110			0.970 ± 0.110				
	NR			NR			P < 0.05				
Joaquin(a) 2010	1173.8 ± 218.7				0.64 ± 0.2			0.68 ± 0.02	0.75 ± 0.02		
	1180.0 ± 191.3				0.63 ± 0.01			0.64 ± 0.01	0.74 ± 0.01		
	NS				NS			P < 0.05	NS		
Joaquin(b) 2010	1121.5 ± 212.7				0.64 ± 0.2			0.63 ± 0.01	0.72 ± 0.02		
	1180.0 ± 191.3				0.63 ± 0.01			0.64 ± 0.01	0.74 ± 0.01		
	NS				NS			P < 0.05	NS		
Tan Keli 2011						1.022 ± 0.085	0.919 ± 0.072		1.189 ± 0.095	1.209 ± 0.116	1.189 ± 0.101
						0.866 ± 0.071	0.860 ± 0.064		1.049 ± 0.097	1.026 ± 0.119	1.054 ± 0.109
						P < 0.05	NR		P < 0.01	P < 0.01	P < 0.01
Yang Zhanguang 2018					1.20 ± 0.14	0.93 ± 0.06					1.34 ± 0.19
					1.06 ± 0.18	0.67 ± 0.07					1.24 ± 0.21
					P = 0.05	P = 0.00					P = 0.04
Li Fengshuan 2019						0.544 ± 0.063	0.531 ± 0.058				
						0.521 ± 0.054	0.514 ± 0.069				
						P = 0.089	P = 0.058				
Kontulainen 1999		430 ± 54	381 ± 45								
		414 ± 53	407 ± 55								
		NR	NR								
Ashizawa 1999		143 ± 6	127 ± 6			432 ± 10	407 ± 12				
		131 ± 8	128 ± 8			409 ± 16	400 ± 19				
		P < 0.05	NS			P < 0.05	NS				
Haapasalo 2000		409.4 ± 63.4	358.9 ± 50.8								
		388.0 ± 53.1	317.0 ± 55.5								
		P < 0.001	NS								

Of the 10 included studies, one was a longitudinal cohort study, while the remaining nine were cross-sectional studies. The characteristics of these eligible studies are summarized in Table 3. Risk of bias assessments using the JBI tool showed that the cross-sectional studies scored between 3 and 6 out of a maximum of 7 (Table 6), while the cohort study received a score of 7 out of 10 (Table 7).

**Table 6:** Overview of the results from the risk of bias assessment using the Johanna Briggs Institute critical appraisal tool for Analytical Cross-sectional Studies.

Study	Item								Total score
	1	2	3	4	5	6	7	8	
Calbet et al. 1998	Y	N	N	N/A	N	N	Y	Y	3
Claudia 2008	Y	Y	N	N/A	N	N	Y	Y	4
Guo Liang 2010	Y	Y	Y	N/A	N	Y	Y	Y	6
Joaquin 2010	Y	Y	Y	N/A	Y	N	Y	Y	6
Tan Keli 2010	Y	Y	Y	N/A	N	Y	Y	Y	6
Yang Zhanguang 2018	Y	Y	Y	N/A	N	N	Y	Y	5
Li Fengshuan 2019	Y	Y	Y	N/A	N	N	Y	Y	5
Haapasalo 2000	Y	Y	Y	N/A	N	N	Y	Y	5
Ashizawa et al. 1999	Y	N	Y	N/A	N	N	Y	Y	5

Y = Yes; N = No. N/A = Not applicable.

**Table 7:** Overview of the results from the risk of bias assessment using the Johanna Briggs Institute critical appraisal tool for Cohort Studies.

study	Item											Total score
	1	2	3	4	5	6	7	8	9	10	11	
Kontulainen et al., 1999	Y	Y	N/A	Y	Y	N	Y	Y	Y	N	N	7

Y = Yes; N = No. N/A = Not applicable.

Among the 10 studies, 3 did not use reliable and valid methods to measure exposure variables, 8 failed to identify confounding factors, 7 did not implement effective measures to address confounding, and 1 did not employ strategies to manage incomplete follow-up. These limitations contributed to a high risk of performance, detection, and attrition bias in several studies.

A sensitivity analysis was also conducted. The results indicated that, after excluding each individual study, the variation in BMC or BMD values did not exceed 15%, and the overall effect size remained stable. This suggests that the results of the meta-analysis are robust and reliable.

## META analysis result

### BMC

#### Whole body BMC.

**Fig 24:**
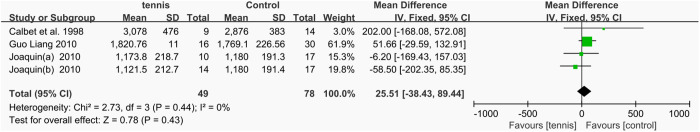
Forest map of the effect of tennis on whole body BMC.

A total of three studies [37,50,51] reported changes in whole-body BMD, involving 127 participants, 49 in the tennis group and 78 in the control group. As no heterogeneity was observed among the studies (I^2^ = 0%, P = 0.44), a fixed-effect model was used for the meta-analysis. The results indicated no significant difference in whole-body BMD between the tennis and control groups (MD = 25.51 mg/mm, 95% CI [−38.43, 85.35], P = 0.43), suggesting that tennis training did not have a statistically significant effect on whole-body BMD (Fig 24).

#### Dominant arm BMC.

**Fig 25:**
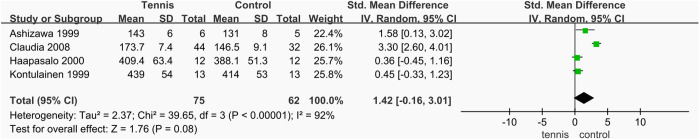
Forest map of the effect of tennis on dominant arm BMC.

A total of four studies [38,55–57] analyzed BMC of the dominant arm, involving 137 participants (75 in the tennis group and 62 in the control group). The heterogeneity test revealed substantial heterogeneity (I^2^ = 92%, P < 0.00001), necessitating the use of a random-effects model for the meta-analysis. The results showed no statistically significant difference in dominant arm BMC between the tennis and control groups (SMD = 1.42 mg/mm, 95% CI [−0.16, 3.01], P = 0.08). However, due to the high level of heterogeneity, the results are inconclusive, and it remains uncertain whether tennis training significantly affects dominant arm BMC. Further analysis is needed to explore the sources of heterogeneity and to clarify the effect of tennis on BMC in the dominant arm (Fig 25).

#### Non-dominant arm BMC.

**Fig 26:**
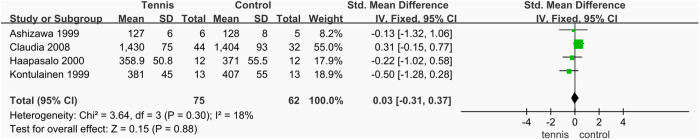
Forest map of the effect of tennis on non-dominant arm BMC.

A total of four studies [38,55–57] analyzed BMC of the non-dominant arm, involving 137 participants (88 in the exercise group and 75 in the control group). As heterogeneity among the studies was low (I^2^ = 18%, P = 0.30), a fixed-effect model was applied for the meta-analysis. The results showed no significant difference in non-dominant arm BMC between the tennis and control groups (SMD = 0.03 mg/mm, 95% CI [−0.31, 0.37], P = 0.88), indicating that tennis training had no significant effect on BMC in the non-dominant arm (Fig 26).

#### Whole body BMD.

**Fig 27:**
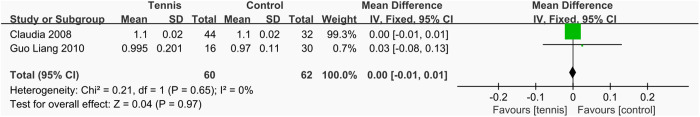
Forest map of the effects of tennis on whole body BMD.

A total of two studies [38,50] examined the effects of tennis on whole-body BMD, involving 122 participants (60 in the tennis group and 62 in the control group). As no heterogeneity was observed (I^2^ = 0%, P = 0.65), a fixed-effect model was applied for the meta-analysis. The results showed that BMD in the tennis group was not significantly different from that in the control group (MD = 0.00 g/cm2, 95% CI [−0.01, 0.01], P = 0.97), indicating that tennis training had no significant effect on whole-body BMD (Fig 27).

### Upper limb BMD

#### Lumbar spine BMD.

**Fig 28:**
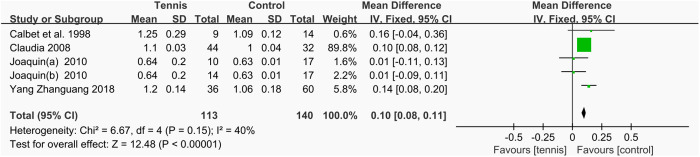
Forest map of the effect of tennis on lumbar spine BMD.

A total of four studies [37,38,51,53], involving 253 participants (113 in the tennis group and 140 in the control group), reported the effects of tennis on lumbar spine BMD. As heterogeneity among the studies was low (I^2^ = 40%, P = 0.15), a fixed-effect model was applied for the meta-analysis. The results showed that lumbar BMD in the tennis group was significantly higher than in the control group (MD = 0.10 g/cm2, 95% CI [0.08, 0.11], P < 0.00001), indicating that tennis training can significantly improve lumbar BMD (Fig 28).

#### Dominant radial and arm BMD.

**Fig 29:**
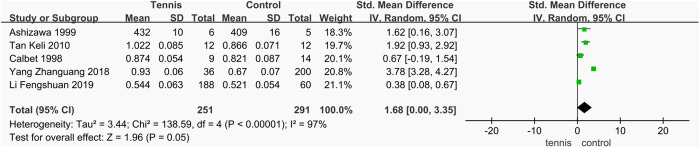
Forest map of the effect of tennis on dominant radial and arm BMD.

A total of five studies [37,46,47,50,51] analyzed the effect of tennis training on BMD of the dominant arm and radius, involving 542 participants (251 in the tennis group and 291 in the control group). The meta-analysis revealed substantial heterogeneity among the studies (I^2^ = 97%, P < 0.00001), prompting the use of a random-effects model. The results showed that BMD of the dominant arm and radius was significantly higher in the tennis group compared to the control group (SMD = 1.68 g/cm^2^, 95% CI [0.00, 3.35], P = 0.05). However, due to the high level of heterogeneity, the reliability of this finding is uncertain, and further investigation is needed to confirm the effect of tennis on dominant arm and radial BMD (Fig 29).

#### Non-dominant arm and radial BMD.

**Fig 30:**
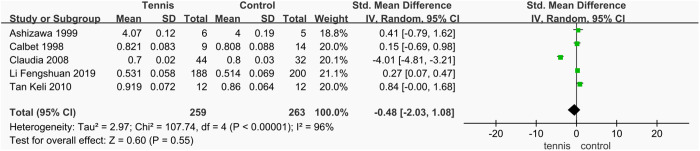
Forest map of the effect of tennis on BMD of non-dominant radial arm.

A total of five studies [37,38,52,54,56] examined the effect of tennis training on BMD of the non-dominant arm and radius, involving 522 participants (259 in the tennis group and 263 in the control group). The meta-analysis revealed high heterogeneity among the studies (I^2^ = 96%, P < 0.00001), and a random-effects model was applied. Compared with the control group, the tennis group showed no statistically significant advantage in non-dominant arm and radial BMD (SMD = −0.48 g/cm^2^, 95% CI [−2.03, 1.08], P = 0.55), indicating that tennis training did not significantly affect non-dominant arm and radial BMD (Fig 30). However, due to the high heterogeneity among the included studies, it is not possible to draw a definitive conclusion about the effect of tennis on non-dominant arm and radial BMD. Therefore, subgroup analysis is warranted to explore potential sources of heterogeneity and better assess the true effect.

### Lower limb BMD

#### Trochanter greater BMD.

**Fig 31:**
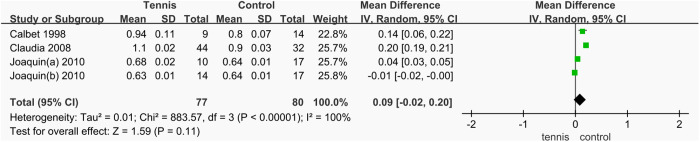
Forest map of the effect of tennis on BMD of the greater trochanter.

A total of three studies [37,38,51] reported changes in BMD of the greater trochanter, involving 157 participants (77 in the tennis group and 80 in the control group). The heterogeneity test indicated extremely high heterogeneity (I^2^ = 100%, P < 0.00001); therefore, a random-effects model was used to combine the effect sizes. The meta-analysis showed no statistical difference in greater trochanter BMD between the tennis and control groups (MD = 0.09 g/cm^2^, 95% CI [−0.02, 0.20], P = 0.11). Due to the high heterogeneity, it is not possible to draw a definitive conclusion regarding the effect of tennis training on greater trochanter BMD. Further subgroup analysis is needed to identify potential sources of heterogeneity (Fig 31).

#### Femoral neck BMD.

**Fig 32:**
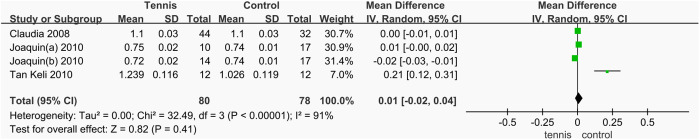
Forest map of the effect of tennis on BMD of femoral neck.

A total of three studies [38,51,52] reported changes in femoral neck BMD, involving 158 participants (80 in the tennis group and 78 in the control group). The heterogeneity test revealed substantial heterogeneity among the studies (I^2^ = 91%, P < 0.00001), prompting the use of a random-effects model to combine the effect sizes. The results indicated no significant difference in femoral neck BMD between the tennis and control groups (MD = 0.01 g/cm^2^, 95% CI [−0.02, 0.04], P = 0.41). Given the high level of heterogeneity, the effect of tennis training on femoral neck BMD could not be conclusively determined. Therefore, subgroup analysis is warranted to explore potential sources of heterogeneity and better clarify the impact of tennis on femoral neck BMD (Fig 32).

#### Left femur BMD.

**Fig 33:**
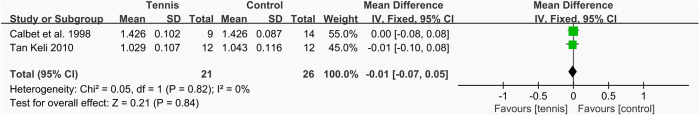
Forest map of the effect of tennis balls on left femur BMD.

A total of two studies [37,52] examined the effects of tennis training on left femur BMD, involving 47 participants (21 in the tennis group and 26 in the control group). A fixed-effect model was used for the meta-analysis, as there was no heterogeneity between the studies (I^2^ = 0%, **P* *= 0.82). The results showed no statistically significant difference in left femur BMD between the tennis and control groups (MD = ^−^0.01 g/cm^2^, 95% CI [^−^0.07, 0.05], **P* *= 0.84), indicating that tennis training did not significantly improve BMD of the left femur (Fig 33).

#### Right femur BMD.

**Fig 34:**
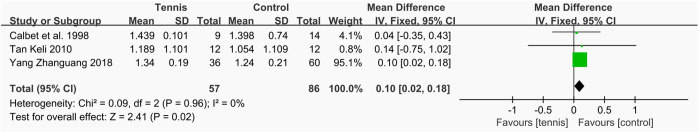
Forest map of the effect of tennis balls on BMD of the right femur.

A total of three studies [37,52,53], involving 143 participants (57 in the tennis group and 86 in the control group), reported the effects of tennis on BMD of the right femur. The meta-analysis using a fixed-effect model showed no heterogeneity among the studies (I^2^ = 0%, **P* *= 0.96). Compared with the control group, the tennis group demonstrated a significant increase in right femur BMD (MD = 0.10 g/cm^2^, 95% CI [0.02, 0.18], **P* *= 0.02), indicating that tennis training can significantly improve BMD of the right femur (Fig 34).

### Subgroup analysis

#### Playing experience.

**Fig 35:**
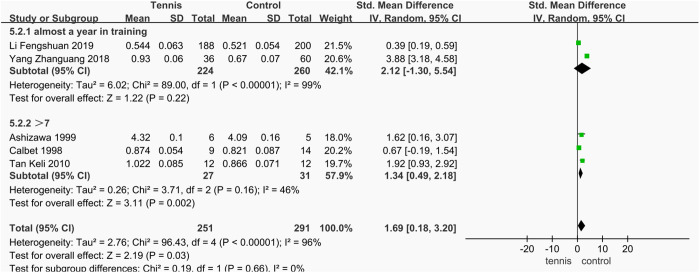
Results of subgroup analysis of dominant arm BMD.

Among the five studies that assessed BMD of the dominant arm and radius, a subgroup analysis was conducted based on training duration and frequency. The combined effect of the three studies in which participants had more than 7 years of tennis experience and trained more than 15 hours per week showed a significant improvement in dominant radial BMD compared to the control group (SMD = 1.34 g/cm^2^, 95% CI [0.49, 2.18], **P* *= 0.002), indicating a statistically significant difference (**P* *< 0.05). In contrast, the combined effect of the studies in which participants had more than 1 year of experience and trained more than 1 hour per day was not statistically significant (SMD = 2.12 g/cm^2^, 95% CI [^−^1.30, 5.54], **P* *= 0.22), suggesting no meaningful difference in dominant radial BMD compared to the control group (P > 0.05). These results suggest that a training history of over 7 years and more than 15 hours per week is associated with a significant improvement in dominant arm and radial BMD in males (Fig 35).

**Fig 36:**
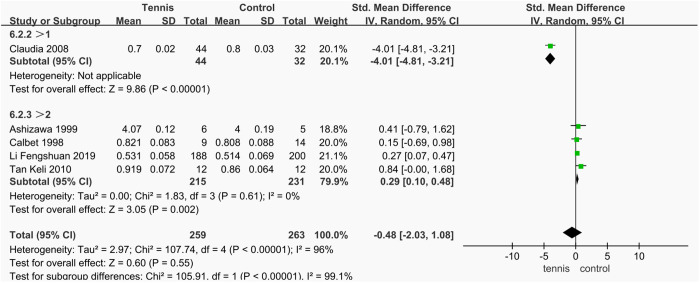
Results of subgroup analysis of non-dominant arm BMD.

Among the five studies that reported BMD in the non-dominant arm, a subgroup analysis was conducted based on training duration. The combined effect of four studies involving participants with more than 2 years of tennis training showed a significant improvement in non-dominant arm BMD compared to the control group (SMD = 0.29 g/cm^2^, 95% CI [0.10, 0.48], **P* *= 0.002). In contrast, the study involving participants with more than 1 year of training reported a large negative effect size (SMD = ^−^4.01 g/cm^2^, 95% CI [^−^4.81, ^−^3.21], **P* *< 0.00001). Despite the contrasting effect directions, both training durations showed statistically significant differences in non-dominant arm BMD compared with the control group (**P* *< 0.05), suggesting that more than one year of tennis training can significantly improve BMD in the non-dominant arm in males (Fig 36).

#### Training volume.

Among the three studies that reported femoral neck BMD, a subgroup analysis was conducted. The combined effect of two studies in the tennis group showed no significant difference compared to the control group (MD = 0.01 g/cm^2^, 95% CI [^−^0.00, 0.01], **P* *= 0.30). In another study that included two groups with different training volumes, the subgroup with training time < 5 hours/week showed an effect size of MD = 0.09 g/cm^2^, 95% CI [^−^0.14, 0.32], **P* *= 0.43.

**Fig 37:**
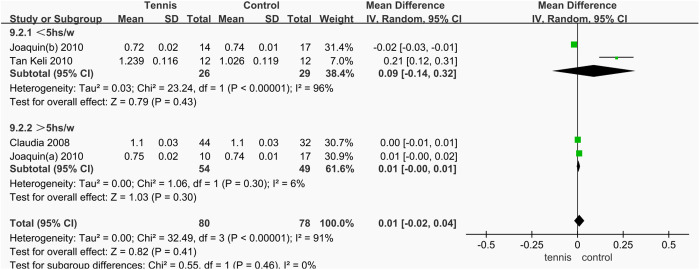
Results of subgroup analysis of femoral neck BMD.

The results of the subgroup analysis indicated that, in both groups, tennis training did not produce a statistically significant improvement in femoral neck BMD compared to the control group (P > 0.05). Therefore, it can be concluded that training volume has little impact on femoral neck BMD in males (Fig 37).

#### Age.

**Fig 38:**
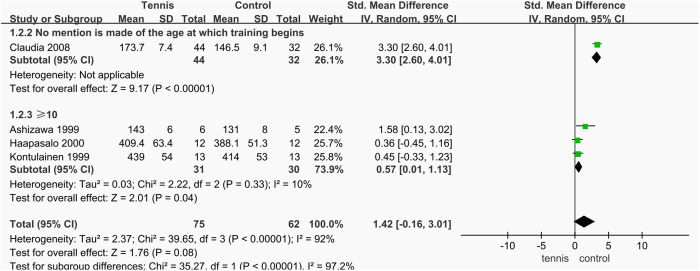
Results of subgroup analysis of Dominant arm BMC.

Among the four studies that reported on dominant arm BMC, one study showed a notably large effect size (SMD = 3.30 mg/mm, 95% CI [2.60, 4.01], **P* *< 0.000001). For the remaining three studies, the combined effect size was SMD = 0.57 mg/mm, 95% CI [0.01, 1.13], **P* *= 0.04, specifically for participants who began tennis training at age 10 or older. Both groups showed statistically significant differences in dominant arm BMC compared to the control group (**P* *< 0.05). However, because the three-study subgroup (excluding Claudia’s study) did not specify the exact age at which participants began training, it is difficult to draw a definitive conclusion. Nevertheless, the results suggest that the impact of tennis on dominant arm BMC may be associated with the age at which training is initiated, with training begun at 10 years or older showing a more pronounced effect (Fig 38).

**Fig 39:**
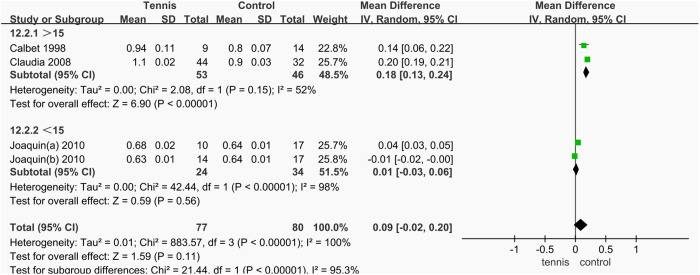
Results of subgroup analysis of greater trochanteric BMD.

Among the three studies that reported greater trochanteric BMD, a subgroup analysis was conducted based on participant age. The combined effect of two studies involving participants over 15 years old showed a significant improvement in greater trochanteric BMD compared to the control group (MD = 0.18 g/cm^2^, 95% CI [0.13, 0.24], **P* *< 0.00001). In contrast, two groups from another study, involving participants under 15 years old, showed no significant difference in BMD compared to the control group (MD = 0.01 g/cm^2^, 95% CI [^−^0.03, 0.06], **P* *= 0.56). These results indicate that tennis training can significantly increase greater trochanteric BMD in males over the age of 15, while no significant effect was observed in those under 15 (Fig 39).

## Discussion

### Effect of tennis on whole body BMD

The results of the meta-analysis indicated that tennis training did not have a significant effect on total body BMC or total body BMD in males. Although the values in the tennis group were slightly higher than those in the control group, the difference was not statistically significant. This may be attributed to the fact that, in an effort to prevent excessive bilateral limb asymmetry, tennis training is often supplemented with additional strength or resistance exercises. These complementary activities may help maintain musculoskeletal balance between both sides of the body, thereby minimizing overall changes in total body BMC and BMD.

### Effect of tennis on upper limb BMD

The results of the meta-analysis showed that the BMC and BMD of the dominant arm were significantly higher in the tennis group than in the control group. These findings are consistent with those of Chapelle et al. [42], who also observed a more pronounced asymmetry in upper limb BMD among male tennis players. Our results further support this conclusion, demonstrating that tennis exerts a significant effect on the BMC and BMD of the dominant arm, with clear asymmetry between the dominant and non-dominant sides.

This asymmetry can be attributed to the unilateral nature of tennis, a sport involving repeated use of a single arm. During play, athletes repeatedly swing the racquet to maintain rally continuity. The combination of racquet weight and incoming ball velocity generates external resistance, requiring muscle contraction and torsional force to counteract these forces [48,49]. As a result, the dominant arm experiences increased mechanical loading. This mechanical stimulus promotes localized BMC accumulation and increases cortical thickness to accommodate the load [58]. Furthermore, the resulting bone mineral accumulation and endosteal remodeling shift the cortical center away from the neutral axis, enhancing bone strength, torsional resistance, and fracture prevention [59].

Additionally, our analysis revealed that more than one year of tennis training led to an increase in BMD in the non-dominant radial arm. This can be explained by the biomechanical demands of the two-handed backhand stroke in tennis. During a backhand swing, both hands grip the racquet and participate in the stroke, subjecting the non-dominant arm to a mechanical load similar to that experienced by the dominant arm. This load induces physiological stress on the non-dominant arm, thereby promoting increased BMD [52].

### Effect of tennis on Lumbar spine BMD

The results of the meta-analysis showed that lumbar spine BMD was significantly higher in the tennis group compared to the control group, indicating that tennis has a positive effect on lumbar BMD. This may be attributed to the biomechanical demands of the sport: during strokes, players must rapidly and forcefully twist their torso, engaging the back muscles and generating substantial pressure on the lumbar spine. In addition, the lumbar region must absorb and stabilize the mechanical loads produced by various tennis techniques and movements [54]. These combined forces promote adaptive changes in lumbar bone density through repetitive loading and muscle contraction.

### Effects of tennis on lower limb BMD

The results of the meta-analysis indicated that tennis has a positive effect on BMD in the lower limbs of males. During play, tennis athletes frequently engage in multidirectional running and abrupt stops to execute return strokes. These movements are often accompanied by trunk rotation, which enhances the speed and power of the return. Such rotation requires strong leg support and force generation. When players push off the ground, a reaction force is generated, increasing the mechanical load on the lower limbs and thereby promoting bone density in the leg bones [53].

Previous research has shown that nearly half of all hip fractures are caused by osteoporosis [60]. As a critical anatomical structure of the hip, the greater trochanter plays an important role in the diagnosis and treatment of femoral neck fractures and hip dislocations. An increase in BMD in this region can strengthen the connection between the hip joint and the femur, stabilizing the joint against external forces and reducing the risk of fracture.

Recent studies have also confirmed that tennis exerts considerable mechanical stimulation on the lower limbs, including the greater trochanter, leading to increased BMD in these regions. Our study further supports this finding, revealing that the impact of tennis on greater trochanteric BMD appears to be age-dependent [52,54]. Specifically, the positive effect is more pronounced in individuals over the age of 15. This age marks late adolescence, a period during which physical maturity and physiological stability are achieved. To enhance performance during this stage, athletes typically undergo more intensive and frequent training, which amplifies mechanical stimulation across the limbs, including the greater trochanter, and promotes increased bone density.

In contrast, our findings suggest that tennis has minimal impact on femoral neck BMD. The femoral neck serves as a connector between the pelvis and the femur. It is possible that mechanical stress during tennis movement is primarily distributed to the femoral shaft and surrounding structures, leaving the femoral neck less directly stimulated by load-bearing forces and thus less affected in terms of bone density gains.

## Limitations and suggestions for future research

This study has several limitations. First, due to the limited number of studies examining the effects of tennis on male BMD, only a small number of relevant articles were included, which may compromise the scientific rigor of the systematic review. Second, some included studies had small sample sizes, potentially resulting in insufficient statistical power. Third, the anatomical sites assessed for BMD varied across studies, complicating data extraction and reducing the consistency and comparability of reported outcomes. Fourth, objective constraints such as language barriers and limited database access may have led to incomplete literature retrieval, potentially affecting the reliability of the meta-analysis results.

In light of these limitations, future research should aim to increase the number of studies focusing on the effects of tennis on male BMD to strengthen the evidence base. Additionally, standardizing anatomical measurement sites would improve the reliability and comparability of findings, while enrolling larger sample sizes would enhance statistical power and the robustness of conclusions.

## Brief summary

This study comprehensively analyzed the effects of tennis on BMD across various regions of the male body. The main findings indicate that tennis has a positive effect on increasing dominant arm BMC, lumbar spine BMD, dominant radial BMD, non-dominant radial BMD, and right femur BMD. Subgroup analysis further revealed that a training duration of at least one year and more than 5 hours per week significantly improves dominant arm BMD, while training for more than 7 years and over 15 hours per week has an even greater impact on both dominant arm and radial BMD. In contrast, tennis had limited or no significant effect on whole-body BMC, whole-body BMD, non-dominant arm BMC, femoral neck BMD, and left femur BMD. Notably, tennis had the strongest effect on greatest trochanteric BMD in males over the age of 15.

## Conclusion

In conclusion, tennis training significantly improves bone health indicators in males, including dominant arm BMC, lumbar spine BMD, dominant radial BMD, non-dominant radial BMD, and right femur BMD. The effects of tennis on BMD are asymmetrical, with more pronounced improvements observed in the dominant limb compared to the non-dominant limb. Additionally, tennis training significantly increases greater trochanteric BMD in males over the age of 15.
