# Correction: The effect of tennis on male bone mineral density: A meta-analysis

**DOI:** 10.1371/journal.pone.0338950

**Published:** 2025-12-12

**Authors:** Huang Huiwen, Huang Jun, Fu Dong, Fu Yan, Wang Tao

The second author’s name is spelled incorrectly. The correct name is: Huang Jun. The correct citation is: Huiwen H, Jun H, Dong F, Yan F, Tao W (2025) The effect of tennis on male bone mineral density: A meta-analysis. PLoS One 20(8): e0328636. https://doi.org/10.1371/journal.pone.0328636

There is an error in affiliation 1 for author Huang Huiwen. The correct affiliation 1 is: Chengdu Sport University, Chengdu, China.
